# Nurse competence provides more individuality in the care of older hospitalized people

**DOI:** 10.1002/nop2.1569

**Published:** 2022-12-26

**Authors:** Lahtinen Katja, Lemetti Terhi, Stolt Minna, Katajisto Jouko, Suhonen Riitta

**Affiliations:** ^1^ Department of Nursing Science University of Turku Turku Finland; ^2^ Department of Social and Health Care City of Helsinki Helsinki Finland; ^3^ Helsinki University Hospital and University of Helsinki Helsinki Finland; ^4^ Statistics Unit, Department of Mathematics and Statistics University of Turku Turku Finland; ^5^ Welfare Services Division Turku University Hospital and City of Turku Turku Finland

**Keywords:** individualized care, nurse competence, older people, patient‐centred care

## Abstract

**Aim:**

The aim of the study was to assess Registered Nurses' perceptions of general nurse competence, patient‐centred care competence, and individuality in the care of older patients and to explore their associations.

**Design:**

A descriptive correlative survey.

**Methods:**

Data were collected using questionnaires at one Finnish university hospital during winter 2016–2017 amongst Registered Nurses (*n* = 223) and analyzedd statistically using descriptive and inferential statistics (ANOVA, Pearson's correlations coefficients) and path analysis.

**Results:**

Registered Nurses assessed their general competence, patient‐centred care competenc,e and individuality in the care of older patients at a good level. The Path model confirmed general nurse competence was a predictor of patient‐centred care competence, which in turn was a predictor of individuality in the nursing care of older patients. The novelty lies in empirical confirmation of the association between nurse competence and individuality in the care. Increasing competence may enhance individuality in the care of older people and enable interventions to support care outcomes.

## 1 INTRODUCTION

Older people with complex health problems are a challenging group of patients in acute care (e.g. Ellis et al., 2017; Goldberg et al., 2014; Yevchak et al., 2017), and warrant specific consideration, for example, a comprehensive geriatric assessment on admission to a hospital (Ellis et al., 2017; Goldberg et al., 2014). Earlier studies have shown that older patients who received individualized care (Spaling et al., 2015; Suhonen et al., 2014) based on a comprehensive geriatric assessment when admitted to hospital (Ellis et al., 2017), achieved better health outcomes (Spaling et al., 2015; Suhonen et al., 2014) and were living in their own homes after discharge (Ellis et al., 2017). However, older patients have often been found to face adverse events or harmful incidents during hospitalization (McGrath et al., 2017) or their care needs are not met and thus there is an increased risk for readmission (Carthon et al., 2017; Kalánková et al., 2021).

Individualized care or patient‐centred care (PCC) are worldwide strategies predicting positive outcomes for patients (Brownie & Nancarrow, 2013; Suhonen et al., 2019). However, individuality and patient‐centredness in care demand special competencies from Registered Nurses (RN) (Hwang, 2015; Jakimowicz & Perry, 2015). Some indications of the association between nurse competence and individualization when providing care for older patients have been stated (Delaney, 2018; Jakimowicz & Perry, 2015), but not empirically verified by modelling. Therefore, we empirically tested the hypothesized associations between general nurse competence, patient‐centred care competence and individuality in the care of older patients. The aim of this study was to assess RNs' perceptions of general nurse competence, patient‐centred care competence, and individuality in the care of older patients, and explore their associations.

### 1.1 Background

Nursing care of older patients requires special competencies from registered nurses (RNs) (Goldberg et al., 2014), especially proactive and advanced strategies to prevent harm (Ellis et al., 2017), ensure dignity (Hall & Høy, 2012), preserve independence, and maintain the ability to function (Goldberg et al., 2014; McGrath et al., 2017) during acute hospitalization. These actions demand patient‐centred care competence (Hwang, 2015) from RNs. However, RNs often make choices in their practice that follow the prevailing standards rather than choose those that advocate for patient‐centred approaches. (Rushton & Edvardsson, 2018). RNs are skilled professionals responsible for delivering comprehensive care to patients (e.g. Goldberg et al., 2014; World Health Organization, 2017). Older patients appreciate the skills of RNs (Nakrem et al., 2011), and consider their competence to be a part of quality care (Edvardsson et al., 2010).

*Professional competence* in nursing refers to the ability of RNs to work in specific healthcare environments, to combine knowledge, skills, values, and attitudes, and implement them in practice (Meretoja et al., 2004). Based on Meretoja's framework nurse competence can be divided into seven categories: (1) helping role, (2) teaching‐coaching, (3) diagnostic functions, (4) managing situations, (5) therapeutic interventions, (6) ensuring quality and (7) work role. The factors associated with nurse competence are education, experience, RN's personal interests, and different contexts of health care (International Council of Nurses, 2013). Nurse competence develops over time (Meretoja et al., 2015), and nurse competence leads to confidence, safe practice, patient‐centredness, and humane care (Smith, 2012).

There are several studies on nurse competence in hospital environments (e.g. Flinkman et al., 2017; Meretoja et al., 2015). RNs have self‐assessed that their competence to be at a good level (Bahreini et al., 2011; Karlstedt et al., 2015; Meretoja et al., 2015). The categories of helping role and diagnostic functions have been assessed to be at a very good level (e.g. Karlstedt et al., 2015; Meretoja et al., 2015) suggesting that RNs consider themselves skilled professionals. The categories of teaching‐coaching and ensuring quality have been assessed as being the lowest (Flinkman et al., 2017). The RNs assessed that when working with older patients, the age, work experience, and education of the RN increased their nurse competence (Karlstedt et al., 2015). RNs working in hospital environments have also assessed that the age and work experience of RNs have a positive influence on nurse competence (Meretoja et al., 2015).

Competence is crucial in providing nursing care for patients (Edvardsson et al., 2010; Goldberg et al., 2014). Whilst the connection between nurses' competence and some patient outcomes, such as quality of care, are well represented in the literature (e.g. Flinkman et al., 2017), empirical evidence on the association between competence and provision of individualized care is very limited, however, it does exist (Hwang, 2015). Individuality in care is highly valued by both the patients and nurses (Suhonen et al., 2019), and the ability to individualize nursing care has been found to associate with the skill mix, competence (Hwang, 2015; Li & Porock, 2014), and longer work experience of nurses (Suhonen et al., 2011); thus, highlighting expertise.

*Patient‐centredness* and the care consequently provided is a core value in care and services (Hwang, 2015; Jakimowicz & Perry, 2015) and competence in nursing (Cronenwett et al., 2007) requiring education of nurses (Hwang, 2015), and such care is part of quality and safe care for patients. Patient‐centredness is often used in reference to patient care in a specific context, for example, organization or a patient group (Mead & Bower, 2000) with the goal of maintaining the patient's functional ability (World Health Organization, 2017). This demands professional skills, holistic approaches, respect for patients and their individual needs, and decisions from nurses (World Health Organization, 2017). With the aim of providing comprehensive nursing care there is a growing interest in investigating the use of patient‐centred care for older individuals in acute care settings (Yevchak et al., 2017). However, barriers have been recognized including, for example, failure to see the patient as an individual and limited time and resources (Yevchak et al., 2017) calling for patient‐centred care competence (PCC) (Hwang, 2015).

*Patient‐centred care competence* (PCC) has been defined in terms of RNs' ability to identify patients values (Delaney, 2018), and skill (Hwang, 2015) in providing for the patients physical, and emotional needs, facilitate shared decision making, treating the patient as an individual, and having a positive attitude towards patient care (Cronenwett et al., 2007; Delaney, 2018; Hwang, 2015) to deliver effective, compassionate, and safe nursing care. PCC competence develops with RNs' education, work experience (Cronenwett et al., 2007), and maturing (Hwang, 2015).

*Individualized care*, defined in terms of perceived individuality in care, is an approach to patient care where patients are seen as unique individuals, being different from others, with individual needs and preferences (Suhonen et al., 2019). In accordance with patient‐centred care, individuality in care for older patients and nurses' role in providing such care for older patients are relevant topics in nursing today (e.g. Ministry of Social Affairs and Health in Finland, 2020; World Health Organization, 2017). Individuality in care is defined as “best practice” or “gold standard” (Ryan & Lauver, 2002) taking into account of individuals' clinical situation, personal life situation, and decisional control over care (Suhonen et al., 2019). Care individualization is based on the patient's personal needs that determine the nursing interventions and nurse–patient interactions (Caspar & O'Rourke, 2008). Because every patient is unique, not all nursing interventions fit for all. However, according to research evidence implementing individuality in care is fragmented (Suhonen et al., 2019). Therefore, an individualized intervention can only develop during a nurse–patient interaction in real time (Lauver et al., 2002). In these complex circumstances, working from general nursing guidelines enables the nursing interventions to evolve in nurse–patient interaction in real time contrary to following the protocols (Lauver et al., 2004).

During the past years, individuality in nursing care has been measured both from the patients' and nurses' perspective, especially in older patients' care environments (e.g. Suhonen et al., 2013). Older patients wish to be treated as unique individuals when hospitalized (Hall & Høy, 2012). From their perspective being able to participate, and make decisions about their own care (Suhonen et al., 2019) is associated with decisional control over care, one component of individuality in care. Older patients wish to receive care that is professionally conducted and safe (Edvardsson et al., 2010; Nakrem et al., 2011). They appreciate nurses treating them with respect, giving them time, understanding them and giving them the opportunity to have conversations (Soares et al., 2019). Respecting the older patients and promoting their dignity during hospitalization are important in care provision (Hall & Høy, 2012). Nevertheless, the busy hospital environment was assessed to be a challenging place to treat older patients, especially individuals with memory disorders (Nilsson et al., 2013). The RNs estimated that they lacked time for the older patients and not knowing them well lead to poorer quality of care (Higgins et al., 2007), less individuality in the care and stress for the RNs (Clissett et al., 2013). When assessing individuality in care from the nurses' point of views (Suhonen et al., 2010), nurses are asked to self‐assess to what extent the care they provided supported the older patient's individuality during the work shifts. Nurses have been found to supported individuality in patient care, but less in the area of patients' personal life situation (Suhonen et al., 2010).

To summarize, studies have clearly shown the need for individualized care for older patients as their personal life history and their complex health problems need individual, comprehensive assessment and care. Consequently, this requires competent professionals with advanced general competence and specialized patient‐centred care competence. However, there is a lack of studies analysing these preliminarily suggested associations. Exploring the associations of the concepts of nurse competence, patient‐centred care competence and nurses' perceptions about individuality in care provided could help understand the importance of nurses' role and their competence in enabling the individualization of nursing care for older patients and thereby increase positive health outcomes in acute situations of older patients.

### 1.2 Aim

The aim of this study was to assess RNs' perceptions of general nurse competence, patient‐centred care competence, and individuality in the care of older patients, and explore their associations.

*Research questions*

- What is the nurses' self‐assessed levels of general competence, patient‐centred care competence, and individuality in care provided for older patients?
- What are the associations, if any between nurses' self‐assessed levels of general competence, patient‐centred care competence, and perceptions of individuality in the care provided?

The following hypotheses were set:

The higher the level of nurses' self‐assessed general competence, the higher the level of patient‐centred care competence.

The higher the level of nurses' patient‐centred care competence, the higher the level of individuality in the care provided.

## 2 METHODS

### 2.1 Design

The study used a descriptive and correlational survey design using questionnaires including three validated instruments for data collection. The study adhered to STROBE guidelines for cohort studies.

### 2.2 Study sample

The study was conducted in the in‐patient wards of one university hospital in Southern Finland between October 2016 and January 2017. A convenience sample of RNs was recruited from the fourteen in‐patient wards of the largest university hospital where the majority of patients were ageing individuals. The sample size required was calculated using the “thumb rule,” for example 5–10 respondents per item for the largest construct being the Nurse Competence Scale with 73 items (365–730) for correlational analysis needed for path analysis (Grove et al., 2013). The inclusion criteria for participants were (1) being an RN, (2) working in an acute care hospital in‐patient unit and (3) nursing older people. The total number of RNs approached was 770, and 223 responded to the survey giving the response rate of 29%.

### 2.3 Data collection

The web‐based (Webropol) survey questionnaires were sent electronically through emails to total sample of nurses via the directors of nursing with an invitation letter giving information about the study; the participants were informed that the study was voluntary, anonymous and confidential. Two reminders were sent to the potential respondents via the directors of nursing, two weeks and four weeks after the initial invitation in October 2016.

### 2.4 Instruments

Three validated instruments were used: The Nurse Competence Scale (NCS; Meretoja, 2003), the Patient‐centred Care Competency Scale (PCCS; Hwang, 2015) and the Individualized Care Scale‐Nurse Part A (ICS‐Nurse‐A; Suhonen et al., 2010).

The Nurse Competence Scale (Meretoja, 2003; Meretoja et al., 2004) is a 73‐item scale that measures nurses' general competence and is divided into 7 subscales. The subscales are: (1) helping role (7 items), (2) teaching‐coaching (16 items), (3) diagnostic functions (7 items), (4) managing situations (8 items), (5) therapeutic interventions (10 items), (6) ensuring quality (6 items) and (7) work role (19 items). The items are rated using the VAS (0–100), 0 being a very low level of competence and 100 a very high level of competence. The frequency, that is how often the items are used in nursing is rated from 0–3 (very seldom‐very often). (Meretoja, 2003; Meretoja et al., 2004) The Nurse Competence Scale has been widely used in international contexts and has been translated into many different languages. The instrument has demonstrated good content validity and internal consistency, the content validity index (CVI) being 0.83 based on experts' ratings and Cronbach's alpha varying at the category level between 0.61 and 0.97 for internal consistency. Structural validity of the NCS is confirmed using principal component analysis (PCA), and the total variance explained was 52.7%. (Flinkman et al., 2017).

The Patient‐centred Care Competency Scale (Hwang, 2015) is a self‐administered tool to measure competence in patient‐centred care. The scale consists of 17 items divided into four different areas: (1) Respecting patients' perspectives (6 items), (2) Promoting patient involvement in care processes (5 items), (3) Providing for patient comfort (3 items) and (4) Advocating for patients (3 items). Self‐assessment is rated by using a Likert‐type 5‐point scale (1 = minimal, 2 = below average, 3 = equals average, 4 = good, 5 = excellent). (Hwang, 2015) The Patient‐centred Care Competency Scale is a relatively new instrument, but has been determined to be a valid and reliable tool (Hwang, 2015). PCA confirmed the four‐factor solution explaining 61.8% of the variance, and the internal consistency was good (Cronbach's alpha =0.92 for total and for sub‐scales 0.80–0.85).

The Individualized Care Scale‐Nurse‐A (Suhonen et al., 2010) was designed to measure nurses' views on how they support patients' individuality through nursing activities, that is individuality in care. The ICS‐Nurse A uses a self‐assessment with a Likert‐type 5‐point scale (Suhonen et al., 2010). The ICS‐Nurse‐A has 17 items in total divided into three sub‐scales. The sub‐scales measure the support nurses provide for the patient's clinical situation (7 items), personal life situation (4 items), and decisional control over care (6 items) (Suhonen et al., 2010). This instrument has proven to be reliable, and valid (Suhonen et al., 2019), and has been used in international contexts. Cronbach's alpha values (ranging from 0.88–0.95) of the ICS‐Nurse part A indicate good internal consistency (Suhonen et al., 2019). PCA confirmed the three‐category structure, explaining 52% of the variance (Suhonen et al., 2010).

Socio‐demographic and work‐related background information was requested from the respondents.

### 2.5 Data analysis

A statistical analysis was performed using SPSS version 22.0 for Windows (IBM SPSS). The descriptive data are presented by frequencies, percentages, means and standard deviations. Sum variables were formed based on the theoretical constructs of the instruments in the manuals. Pearson's correlational coefficient (sig two‐tailed) was used to analyse the degree of the correlation between the main sum variables. The associations between the sum variables and background variables were computed using a one‐way analysis of variance (ANOVA). Multiple comparisons were computed using Sidak's test. Cronbach's alpha coefficient was used to measure the internal homogeneity of the instruments and their parts. Mplus version 7.11 (Muthén & Muthén, 2007, Maximum Likelihood procedure) was used to determine the best model for analysing the hypothesized associations. Firstly, the model fit was tested using the chi‐square test of model fit, with degrees of freedom (df) and p‐value, followed by the comparative fit index (CFI, with the criterion of CFI ≥0.95, Hu & Bentler, 1999); the Tucker–Lewis Index (TLI, with the criterion of ≥0.95); the root mean square error of approximation (RMSEA, with the criterion of <0.06, Hu & Bentler, 1999, or a stringent upper limit of 0.07, Steiger, 2007) and the standardized root mean square residual (SRMR; criterion <0.08). Secondly, parameter estimates were calculated and represent the strength of the path between two variables (standardized and unstandardized regression coefficients, standard error SE, *p*‐value). An *R*‐square was used to evaluate the variance each variable explained in the model.

## 3 RESULTS

### 3.1 Respondents

The respondents (*n* = 223) were Registered Nurses. The mean age of the respondents was 39 years (SD 11.6, range 24–64). Most of the respondents were women (93%). The mean length of work experience in health care was 13 years (SD 10, range 0.5–40 years). The mean length of work experience at the unit where the respondent was currently working at the time of the study was 7.5 years (SD 5, range 1 month – 35 years). Most of the respondents were working full time (92%) (Table 1).

**TABLE 1:** Respondents' characteristics

	*n* ^a^	%	Mean (SD)	Range
Age	215		39 (11.6)	23–64
Length of work experience in health care (years)	223		13 (9.9)	0.5–40
Length of work experience in current unit (years)	223		7.6 (7.3)	0–35
Nature of employment	195			
Full time	182	92		
Part‐time	13	7		
Gender	221			
Female	206	93		
Male	15	7		
Education	223			
RN Baccalaureate	152	68		
RN college‐level education	58	26		
Master's degree in nursing	4	2		
Postgraduate classes	9	4		

^a^ *n* changes due to missing responses.

### 3.2 General competence

The general nurse competence was assessed to be at a good level (mean VAS 70.7). From the seven competencies, helping role (mean VAS 76.7) was assessed to be at a very good level (>VAS 75). Teaching‐coaching (mean VAS 69.9), diagnostic functions (mean VAS 73.6), managing situations (mean VAS 71.5), and work role (mean VAS 69.8) were assessed to be at a good level (VAS 50–75). Therapeutic interventions (mean VAS 65.5), and ensuring quality (mean VAS 67.8) were assessed as being lower, but still at a good level (Table 2).

**TABLE 2:** Descriptive results on sum variables

	Mean (SD)	Cronbach's alpha
Nurse competence (NCS, VAS 0–100)	70.7	0.98
Helping role	76.7 (12.9)	0.82
Teaching‐coaching	69.9 (16.8)	0.93
Diagnostic functions	73.6 (15.5)	0.83
Managing situations	71.5 (17.0)	0.88
Therapeutic interventions	65.5 (20.4)	0.92
Ensuring quality	67.8 (17.8)	0.81
Work role	69.8 (17.1)	0.92
Patient‐centred care competence (PCC, 1–5 Likert)	4.04 (0.46)	0.93
Respecting patients' perspectives	4.13 (0.49)	0.84
Promoting patient involvement in care processes	3.78 (0.56)	0.85
Providing for patient comfort	4.31 (0.56)	0.85
Advocating for patients	4.03 (0.56)	0.78
Individuality in care (ICS‐A, 1–5 Likert)	4.32 (0.49)	0.92
Patient's clinical situation	4.61 (0.48)	0.89
Personal life situation	3.88 (0.67)	0.81
Decisional control over care	4.33 (0.55)	0.83

The respondents' education positively correlated with general nurse competence. Furthermore, the categories of teaching‐coaching, managing situations, and therapeutic interventions had an association with education. Age had a positive association with managing situations, therapeutic interventions, ensuring quality, and work role. Men and older nurses assessed managing situations higher than women and younger nurses. Longer work experience in health care and current unit was associated with work role (Table 3).

**TABLE 3:** Background factors in association with the study variables

Scale variable	Age^a^		Gender^a^		Education^a^		Length of work experience in health care^a^		Length of work experience in current ward^a^		Nature of employment^a^	
	*F*	*p*	*F*	*p*	*F*	*p*	*F*	*p*	*F*	*p*	*F*	*p*
NCS total	7.411	0.082	1.313	0.254	2.541	0.007*	0.883	0.349	1.935	0.166	0.005	0.945
Helping role	3.531	0.839	0.109	0.742	2.620	0.076	0.002	0.965	0.003	0.959	0.041	0.062
Teaching‐coaching	3.139	0.078	1.316	0.253	3.278	0.040*	0.001	0.970	3.429	0.066	0.352	0.554
Diagnostic functions	1.010	0.316	0.382	0.537	2.403	0.093	0.054	0.817	0.983	0.323	0.791	0.375
Managing situations	4.916	0.028*	4.778	0.030*	3.134	0.046*	0.324	0.570	1.898	0.170	0.066	0.797
Therapeutic interventions	6.821	0.043*	2.147	0.145	3.214	0.043*	1.807	0.181	1.036	0.310	0.092	0.762
Ensuring quality	10.653	0.001*	0.162	0.688	1.627	0.199	1.119	0.292	0.085	0.770	0.229	0.633
Work role	13.849	0.000*	1.993	0.160	1.693	0.187	6.126	0.014*	2.152	0.014*	0.508	0.477
PCC competence total	0.697	0.129	0.270	0.604	2.075	0.405	0.000	0.988	2.050	0.154	1.561	0.213
Respecting patients' perspectives	0.107	0.469	0.007	0.852	0.728	0.030*	0.444	0.141	0.348	0.192	1.148	0.018*
Promoting patient involvement in care processes	0.047	0.828	0.004	0.948	2.439	0.090	0.423	0.516	1.058	0.305	0.333	0.565
Providing for patient comfort	2.442	0.120	1.823	0.179	0.575	0.563	1.831	0.457	0.555	0.178	2.564	0.111
Advocating for patients	8.353	0.004*	0.111	0.739	3.870	0.023*	3.039	0.083	0.757	0.385	0.963	0.328
ICS‐A	0.799	0.001*	2.259	0.135	7.007	0.373	0.202	0.654	0.466	0.496	1.854	0.175
Patient's clinical situation	0.007	0.935	0.019*	0.889	4.068	0.019*	0.604	0.438	1.104	0.295	0.595	0.442
Personal life situation	0.077	0.782	4.156	0.043*	10.316	0.001*	0.416	0.520	2.076	0.151	0.000	0.998
Decisional control over care	7.718	0.018*	6.307	0.013*	4.106	0.018*	6.085	0.015*	0.633	0.427	6.304	0.013*

^a^ Multi‐factor analysis of variance.

* *p* < 0.05.

### 3.3 Patient‐centred care competence

The RNs assessed their patient‐centred care competence to be at a high level (mean 4.04, SD = 0.46). Of the four subscales, providing for patient comfort was assessed to be at a high level (mean 4.31, SD = 0.56). Promoting patient involvement in care processes was assessed as being the lowest (mean 3.78, SD = 0.56). Respecting patients' perspectives (mean 4.13, SD = 0.49) and advocating for patients (mean 4.03, SD = 0.56) were assessed to be at a high level (Table 2).

RNs' age associated with advocating for patients. Part‐time employees respected the patients' perspective more than full‐time employees. Education had a positive association with respecting patients' perspectives, and advocating for patients (Table 3).

### 3.4 Individuality in care

The RNs assessed that they supported older patients' individuality to a large extent (mean 4.32, SD = 0.49). From the three subscales, the patient's clinical situation was supported to the greatest extent (mean 4.61, SD = 0.48), followed by decisional control over care (mean 4.33, SD = 0.55). However, personal life situation was less supported (mean 3.88, SD = 0.67) (Table 2).

The RNs' education had an association with the support for individuality. Patient's personal situation had an association with gender (female) and education. Factors associated with the perceptions of patient's decisional control were female gender, education, shorter work experience in health care, and part‐time employment. Those with part‐time employment assessed the patient's decisional control higher than those RNs in full‐time employment. The RNs with longer work experience assessed the patients' decisional control lower than RNs whose work experience was shorter (Table 3).

### 3.5 Associations between the variables and modelling

Pearson's correlation coefficients showed statistically significant associations between the NCS total and PCC total (r = 0.350, *p* = <0.001) and between the PCC total and the ICS‐Nurse‐A (*r* = 0.487, *p* < 0.001). The association between the NCS‐total and the ICS‐Nurse‐A was not statistically significant (*r* = 0.103, *p* = 0.195). However, correlation analyses provided evidence on continuing with path analysis to further investigate the nature of the associations.

Path analysis was used to test the two hypothesis sets for relationships. The chi‐square test for model fit showed a good fit (*χ* ^2^ = 0.955, (df 1), *p* = 0.3285). Other fit indices also supported a good fit: comparative fit index CFI 1.00 (within the criterion of CFI ≥0.95); the Tucker‐Lewis Index of 1.002 (TLI, with the criterion of ≥0.95); the root mean square error of approximation RMSEA 0.00 (*p*‐value 0.422) (90% CI 0.00, 0.20; with the criterion of <0.06) and the standardized root mean square residual 0.021(SRMR; criterion <0.08).

The first predictor of interest was general competence which was expected to be a positive predictor of patient‐centred care competence (H1). Second, the mediator patient‐centred care competence was expected to predict perceptions of support for individuality in the care provided/care provision (H2).

**FIGURE 1:**
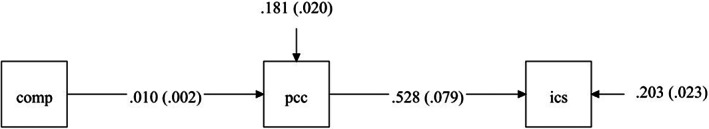
Path model

Figure 1 outlines the findings for the direct effects. Accordingly, general competence positively predicted patient‐centred care competence (*β* = 0.01, S.E 0.002, *t* = 4.785, *p* < 0.001), supporting H1. Furthermore, patient‐centred care competence predicted perceptions of support for individuality in the care provided/care provision (*β* = 0.528, SE 0.079, *t* = 6.684, *p* < 0.001).

## 4 DISCUSSION

This study demonstrated the association between general nurse competence and individuality in older patients' care. This result is novel and supported the hypotheses set based on some very preliminary results (McCormack & McCance, 2006; Meretoja et al., 2015). Self‐assessed general competence, and patient‐centred care competence were at a high level, and perceptions of individuality in the care provided was also assessed as being supported to a large extend. Several studies regarding the main concept have been conducted, but this study advanced the understanding of the underlying associations between the concepts, especially in the care of older patients. Competence and skill mix have been stated to have an impact on nurses' ability to individualize care (Hwang, 2015) and the findings of this study confirm earlier results.

The results showed that RNs' competence supports individualization of care, mediated by the specific patient‐centred care competence. The finding is important as the investment in developing competence impacts on the care outcome (Suhonen et al., 2019). Furthermore, the association between general nurse competence and patient‐centred care competence is fundamental, as it suggests the increase in general competence may support specific competence and through that may improve individuality in the care of older patients. Whilst the data are not recent, research results are important, especially the identified connection between competence and individualized care. Given literature still today suggests needs for development of individualized care for older patients (Suhonen et al., 2019). Overall, general nurse competence was assessed to be at a good level, as was also shown in previous studies conducted in acute hospitals (e.g. Flinkman et al., 2017) and older patients' care environments (Karlstedt et al., 2015). RNs assessed ethical values, individuality in patient care, patient and family empowerment, ability to make decisions about care, and manage different situation important in their work. These are competence categories related to nursing care activities, but categories that were assessed lower were related to developing evidence‐based practices and ensuring quality (Flinkman et al., 2017; Meretoja et al., 2015). This demands educational interventions for nurses (Bahreini et al., 2011), and according to Karlstedt et al. (2015) RNs should have the opportunity to take a postgraduate specialized education in their special area of work, for example care of older patients.

Patient‐centred care competence was assessed as being at a high level. This is an important finding, since patient‐centred care competency leads to individuality in patient care (Hwang, 2015). Providing for patients' comfort can help older patients to recuperate, and respecting patients' perspectives can be seen as a part of good quality care (Hall & Høy, 2012). Advocating for patients is an essential part of caring, especially in acute hospital settings (Canzan et al., 2014), where the busy hospital environment can be seen as a challenge when taking care of older patients (Nilsson et al., 2013) with cognitive impairment (Clissett et al., 2013). Promoting patient involvement in care processes was assessed significantly lower than providing for patients' comfort, respecting patients' perspectives, and advocating for patients. However, promoting older patients to participate in their care leads to individuality in care (Suhonen et al., 2019) and re‐establishes the older patients' dignity (Hall & Høy, 2012).

The RN's self‐assessment was that they support older patients' individuality to a large extent. This can be seen as a positive attitude towards individuality in the care of older patients. Knowing the patient's clinical situation is an important factor from the older patients' perspective, and creates feelings of trust towards the nurses (Canzan et al., 2014; Nakrem et al., 2011; Soares et al., 2019). Giving decisional control, and involving the older patients in their own care creates an interactive relationship between the RN and the older patient (Bedin et al., 2013) and can be seen as a part of quality in care (Hall & Høy, 2012). However, the sub‐scale of knowing the patients and their personal background was less supported than the other sub‐scales. This can lead to poorer quality in care (Higgins et al., 2007), and care needs not being met (Kalánková et al., 2021). According to earlier findings, getting to know the patients and their personal backgrounds also depends on the RN's personal qualities, and their skills when interacting with patients and their acknowledgement of individuality in care (Li & Porock, 2014).

From the background factors, education had an association with general nurse competence and individuality in older patients' care. However, based on earlier studies education increases general nurse competence (Flinkman et al., 2017). The question of age was associated with a higher competence in the categories of managing situations, therapeutic interventions, ensuring quality, and work role. An ageing nursing workforce (Graham & Duffield, 2010) sets demands for proactive strategies from nursing management to support the role of the senior RN (Uthaman et al., 2016), as there is a shortage of RNs globally and a need for competent nurses in health care (World Health Organization, 2017). In addition, the impact of specialized continuing education must be considered in order to ensure RNs' competence when taking care of older patients in an individual manner (Karlstedt et al., 2015; Meretoja et al., 2015).

### 4.1 Limitations

There are some limitations to this study. This was the first study where these three instruments were used together, and these provided further evidence of the associations between the concepts studied. All the instruments were validated and used in international contexts (Flinkman et al., 2017; Hwang, 2015; Suhonen et al., 2019). This study was based on self‐assessment, which can explain some of the positive results in general nurse competence and individuality in the care of older patients. It can be argued that the RNs, who participated in this study, had positive attitudes on the subjects or that those who did not participate, did not see the relevance of the topic or its impact for their work. The study yielded a low response rate of 29% using two reminders. The reminders resulted in a slight increase in respondents to the questionnaires. The university hospital is active in research initiatives, so there may have been other surveys at the same time limiting participation. It is also possible that the questionnaire was considered too long (Grove et al., 2013). The mean age of the respondents was 39, which is lower than the RNs' mean age (44 years) in Finland, but gender distribution equals the nation's gender distribution in RNs (Finnish Nurses Association, 2020). Sample size was estimated for correlation analysis, and the theoretical construct of the instruments. The sample remains limited but offered still valid analysis for path modelling. Furthermore, the data were collected from one university hospital's in‐patient wards, and the response rate was low, so the findings are not generalizable to a wider context.

## 5 CONCLUSIONS

The path model confirmed the connections between general nurse competence and individuality in older patients' care through patient‐centred care competence. Interventions to support individuality in care can possibly benefit from an increase in competence and similar elements. This finding is novel, and can advance in developing the individualization of care, much needed in the care of older patients as well as other patient groups.

These findings can be used in educating registered nurses about the importance of general competence and especially patient‐centred care competence in developing nursing care for older patients (Hwang, 2015), and understanding the importance of knowing the older patient as individuals (Suhonen et al., 2019). In the future, it would be important to examine the perceptions of nurses and older patients simultaneously in order to assess patient outcomes.

## AUTHOR CONTRIBUTIONS

The first author (KL) has acted as the primary researcher in this study and has contributed to this research management, data collection, analysis and drafting and editing the manuscript in entirety. Authors TL, MS and RS have contributed to data analysis, revising, editing and approving the manuscript. Author RS has also contributed to study conception. Author JK has contributed to data analysis and acted as the statistician in this study. All authors have agreed on the final version of and meet at least one of the ICMJE (http://www.icmje.org/recommendations/browse/roles‐and‐responsibilities/defining‐the‐role‐of‐authors‐and‐contributors.html) criteria: Substantial contributions to the conception or design of the work; or the acquisition, analysis, or interpretation of data for the work, drafting the work or revising it critically for important intellectual content and final approval of the version to be published.

## FUNDING INFORMATION

This research received no specific grant from any funding agency in the public, commercial or not‐for‐profit sectors.

## CONFLICT OF INTEREST

No conflict of interest has been declared by the authors.

## ETHICS STATEMENT

The study was approved by the ethical board of the university (REDACTED) and permission to collect the data was obtained from the university hospital (REDACTED). The invitation to attend the study was sent to the Registered Nurses by a third party (directors of nursing) to ensure the anonymity of the respondents and confidentiality of the responses. The returned responses were considered as informed consents to voluntarily participate in the study. No identifying information was collected.

## Data Availability

The data that supports the findings of this study are available from the corresponding author upon reasonable request.
